# Revising with Humans and AI: Effects of Teacher-, GenAI-, and Blended-Feedback on EFL Writing Outcomes

**DOI:** 10.3390/bs16101837

**Published:** 2026-10-08

**Authors:** Feifei Han, Zehua Wang

**Affiliations:** 1 Griffith Institute for Educational Research, Level 2, Macrossan Building (N16), Nathan Campus, Griffith University, Brisbane, QLD 4111, Australia; 2 School of Foreign Languages, Shaanxi Xueqian Normal University, Shenhe 2nd Road, Changan District, Xi’an 710100, China; zehuawang@snsy.edu.cn

**Keywords:** teacher-feedback, GenAI-feedback, blended-feedback, FL writing, revision in FL writing, motivation in FL writing, feedback perception

## Abstract

This mixed-methods, intact-class quasi-experimental study compared teacher-feedback, GenAI-feedback, and teacher–GenAI-blended-feedback in supporting Chinese EFL learners’ writing outcomes. A total of 109 first-year university students from three intact College English classes participated in a six-week intervention. Quantitative outcomes included performance on two revision tasks, performance on a new post-intervention narrative-writing task, writing motivation, and self-efficacy; open-ended questionnaires examined learners’ perceptions of teacher- and GenAI-feedback. Baseline-adjusted MANCOVA showed a significant multivariate effect of feedback type on writing performance, Pillai’s Trace = 0.281, F(6, 208) = 4.90, *p* < 0.001, partial η^2^ = 0.140. GenAI-feedback was associated with stronger immediate revision performance: on the first revision, the GenAI-feedback group outperformed the teacher-feedback group (d = 0.96, *p* < 0.001) and the blended-feedback group (d = 0.55, *p* = 0.046); on the second revision, both the GenAI-feedback and blended-feedback groups outperformed the teacher-feedback group (ds = 0.58 and 0.49, *p*s = 0.028 and 0.047, respectively). In contrast, on the new post-intervention writing task, the teacher-feedback group outperformed both the GenAI-feedback group (d = 0.59, *p* = 0.026) and the blended-feedback group (d = 0.81, *p* = 0.001). Feedback type did not significantly affect motivation or self-efficacy, Pillai’s Trace = 0.008, F(4, 208) = 0.21, *p* = 0.935, partial η^2^ = 0.004. Qualitatively, learners valued teacher-feedback for its clarity, personalization, and emotional support, whereas GenAI-feedback was valued for its efficiency, comprehensiveness, and consistency. These findings point to different short-term strengths of the two feedback approaches: GenAI-feedback was associated with stronger performance during immediate revision, whereas teacher-feedback was associated with stronger performance on a subsequent writing task. The findings suggest that pedagogical uses of GenAI-feedback should consider how its efficiency and comprehensiveness can be integrated with teacher-guided interpretation, reflection, and interpersonal support.

## 1. Introduction

Writing is an essential life skill, while failure to learn to write is associated with poor physical and mental health, participation in crime, welfare dependency, and reduced longevity (Cree et al., 2022). However, writing in one’s first language is already a highly complex and cognitively demanding process, let alone writing in a foreign language (FL) (Hyland, 2019). FL writing requires learners to coordinate multiple cognitive processes simultaneously, including viewpoint generation, organization, language coding, rhetorical planning, monitoring and revision. For learners of English as a foreign language (EFL), limited language resources, uncertainty about genre norms, and the dual need to modify meaning and language form further intensify these requirements.

To help FL writers to improve their writing skills, an essential strategy is to provide high-quality feedback (Fleckenstein et al., 2023). In traditional EFL writing instruction, teacher-feedback plays a core role because teachers can identify learners’ writing problems, provide corrective and explanatory comments, and link feedback to course objectives, classroom teaching, and students’ language proficiency. Therefore, teacher-feedback may not only support immediate text revisions but also promote learners’ understanding of writing principles, which may be applied to subsequent writing tasks. However, providing personalized and constructive writing feedback to students “can be both time-consuming and mentally exhausting” for FL instructors (Teng, 2024, p. 1). In particular, in the context of learning English as a foreign language (EFL), the size of a typical EFL class is often large, and providing individual writing feedback on a regular basis poses a great challenge to EFL instructors (Meyer et al., 2024). These restrictions have prompted teachers and educators to turn to technology-supported feedback systems as a supplementary form of writing support.

The emergence of large language models (LLMs) and generative artificial intelligence (GenAI), such as ChatGPT-4, offers a promising alternative to providing feedback. GenAI-feedback can be generated quickly and cover multiple dimensions of writing, including grammar, vocabulary, organization, coherence, content development and writing norms. Such feedback may be particularly useful during the revision stage, as learners can obtain immediate, multidimensional suggestions to improve their existing drafts. Therefore, recent studies have begun to examine the potential of GenAI-feedback in FL writing contexts (Escalante et al., 2023; Meyer et al., 2024; Teng, 2024). However, newly emerged evidence also indicates that GenAI’s feedback is not without problems. Learners may find the comments generated by AI tools too general, overly complex in language, or not sensitive enough to their language proficiency. They may also passively accept AI suggestions without fully understanding or internalizing the writing knowledge behind them. Therefore, although GenAI-feedback may promote immediate improvement of the text, it remains unclear whether it can support learners’ subsequent independent writing performance to the same extent as teacher-feedback.

Another limitation of the existing literature is that teacher-feedback and GenAI-feedback are typically examined as independent or competing sources, while how the two function in combination within a single instructional sequence has received less attention. From a cognitive perspective, the two may provide different forms of scaffolding. Teacher-feedback can help learners prioritize key writing issues, interpret comments in light of their current proficiency, and link revisions to classroom instruction, whereas GenAI-feedback can provide rapid and comprehensive input across multiple writing dimensions. Therefore, the teacher–GenAI-blended-feedback model may offer a promising feedback approach in EFL writing instruction.

In the present study, blended-feedback refers to the alternating use of these two sources across successive feedback cycles: learners received teacher-feedback on one essay and GenAI-feedback on a subsequent essay. The two sources were never provided on the same draft. This arrangement allows each source’s contribution to a given essay to be identified while still exposing learners to both over the course of the intervention.

Alternation may support revision in two ways. Cognitive load theory holds that working memory is limited, and that material presenting many interacting elements at once imposes load competing with the processing required for learning (Sweller et al., 2019). Comprehensive AI-generated feedback creates this situation, since learners must determine unaided which of many flagged problems are consequential. Teacher-feedback in an earlier cycle may establish priorities and evaluative criteria that learners carry forward, so that extensive GenAI-feedback on a later essay is processed against an existing framework rather than evaluated from scratch. In any single cycle, learners in the blended-feedback condition receive the same amount of feedback as those in the single-source conditions. Alternation therefore cannot reduce the load of a given cycle; its potential benefit lies instead in the priorities and criteria it carried forward to the following cycle.

Alternation may also reduce the risk of passive reliance on GenAI. When GenAI is the sole source across repeated cycles, learners may come to treat AI-generated suggestions as ready-made solutions, implementing changes without understanding why they are needed, an overreliance concern raised across the AI-in-education literature (Kasneci et al., 2023; Tlili et al., 2023). Prior experience of teacher-feedback may provide a reference point against which AI suggestions are judged, encouraging learners to evaluate, interpret, and selectively apply them rather than simply accept them, which supports the evaluative judgment underlying feedback literacy (Carless & Boud, 2018). The blended model therefore holds theoretical promise not merely because it draws on two sources, but because the two support different aspects of writing development: teachers provide contextualized, level-appropriate, and emotionally supportive scaffolding, while GenAI offers timely, comprehensive, and consistent textual support.

Before appropriate approaches to integrating LLMs into EFL writing feedback can be designed, however, several issues warrant empirical investigation. First, although previous studies have examined teacher-feedback or GenAI-feedback in FL writing, few have directly compared teacher-feedback, GenAI-feedback, and blended-feedback within a single design. Second, existing research has focused largely on revision performance or learner perceptions, and little is known about whether feedback sources differ in their effects on immediate revision and on performance in subsequent writing tasks. This distinction matters because successfully revising an existing draft does not necessarily indicate that a learner has acquired writing knowledge that can be applied to new tasks. Third, the motivational consequences of feedback source remain underexplored. Cognitive benefits during revision do not automatically translate into motivational ones, particularly where feedback is static, difficult to interpret, or lacking in interactivity. Finally, more evidence is needed from EFL contexts, in which large class sizes, examination-oriented instruction, and limited access to interactive GenAI tools may shape how students receive and use different forms of feedback.

To address these gaps, the present study examined the effects of teacher-feedback, GenAI-feedback, and blended-feedback on the writing outcomes of Chinese EFL learners. It considered cognitive outcomes (essay revision and post-writing performance) and motivational outcomes (writing motivation and self-efficacy) within a single design, and it also examined the strengths and limitations learners perceived in teacher-feedback and GenAI-feedback, in order to understand how they experienced each source. By comparing three feedback conditions in an intact-class quasi-experimental design, the study aims to provide a more fine-grained account of the distinct pedagogical roles that three types of feedback may play in EFL writing instruction.

Specifically, the study addressed the following three research questions:

(1) To what extent did essay revision and post-writing performance in FL writing differ among Chinese EFL learners receiving teacher-feedback, GenAI-feedback, and teacher–GenAI-blended-feedback?

(2) To what extent did motivation and self-efficacy in FL writing differ among Chinese EFL learners receiving teacher-feedback, GenAI-feedback, and teacher–GenAI-blended-feedback?

(3) How did Chinese EFL learners perceive the strengths and limitations of teacher-feedback and GenAI-feedback?

## 2. Theoretical Framework and Literature Review

### 2.1. Theoretical Framework

**Figure 1:**
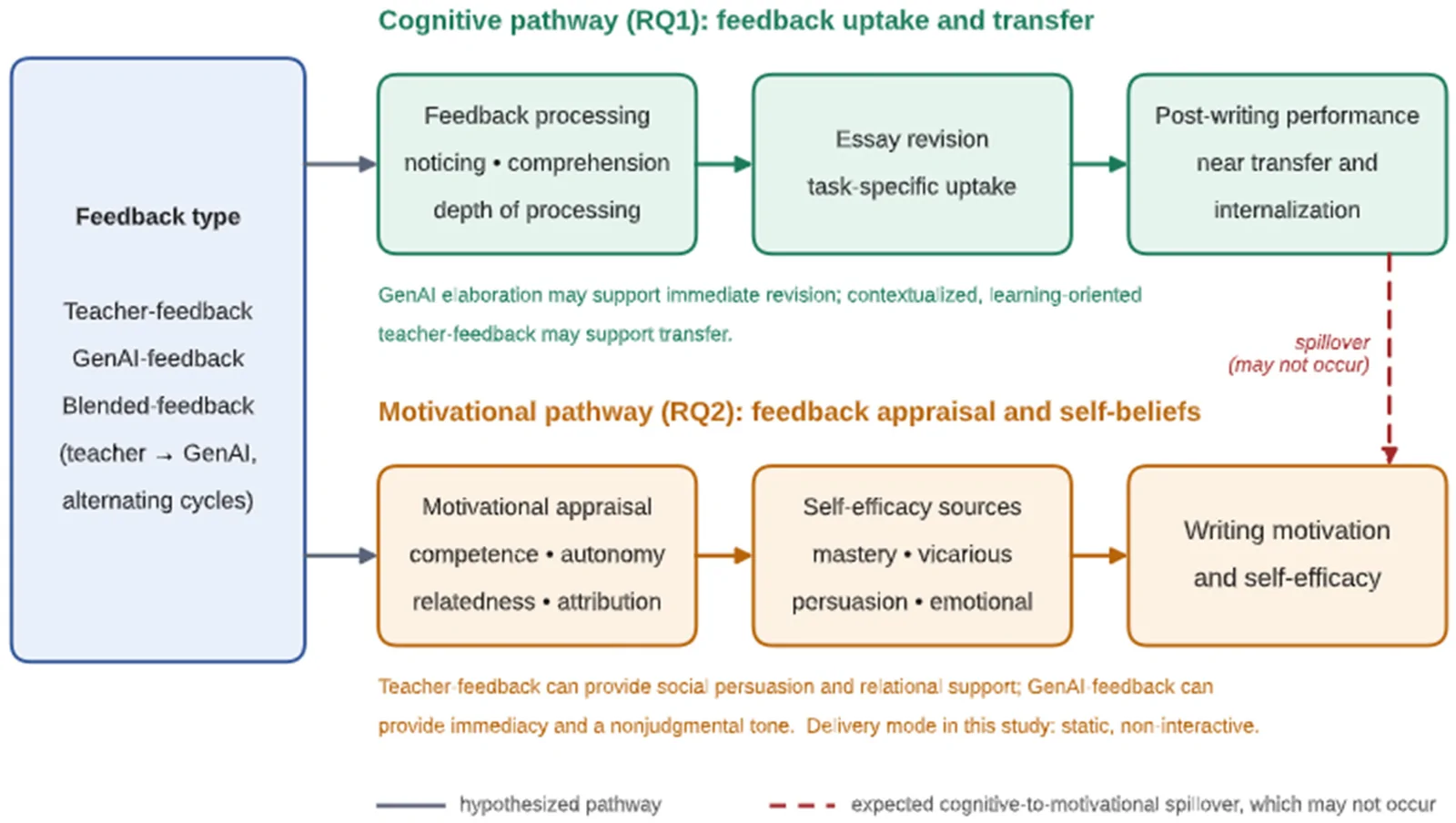
An integrated conceptual model of feedback-type effects on EFL writing.

The following integrated theoretical synthesis brings the two feedback sources together in a dual-pathway model (Figure 1). It links written corrective feedback (WCF) theory, social cognitive accounts of writing self-efficacy, and recent GenAI frameworks in L2 writing.

WCF traditionally regards feedback as an input, guiding learners to focus on the mismatch between their current writing and the target norms. Its effectiveness depends on whether learners notice the marked problems, understand the feedback content, and process the feedback deeply enough to correct their internalized knowledge (Bitchener & Ferris, 2012; Ferris, 2012; Nassaji & Kartchava, 2021). Crucially, this tradition distinguishes between two levels of results: the accuracy of the modification of the received feedback text, which reflects the task-specific acceptance situation and the performance on new writing tasks which reflects transfer and internalization (Karim & Nassaji, 2020). Since transfer requires deeper processing than modifying known text, those feedback features that can maximize immediate acceptance (e.g., coverage, detail and visibility) may not maximize transfer, as transfer relies more on understanding, reflection and linking feedback with generalizable writing knowledge (Hyland & Hyland, 2006; Karim & Nassaji, 2020). This distinction constitutes the cognitive dimension of the model: the feedback type is expected to influence the essay revision and post-writing performance through the depth and quality of feedback processing.

Social cognitive theory complements this explanation by clarifying how feedback shapes learners’ beliefs about their own writing abilities and their willingness to devote themselves to writing efforts. Writing self-efficacy, defined as an individual’s judgment of their own ability to complete writing tasks, develops mainly through four sources: mastery experiences, vicarious experiences, social persuasion, and physiological and emotional states (Bandura, 1997). Feedback is a direct channel for social persuasion and an information clue for ability assessment: encouraging, learner-level, and specific subsequent step-related comments can enhance self-efficacy and motivation, while negative, overly critical, or overwhelming feedback may weaken them (Graham, 2018; Ruegg, 2018; Zarrinabadi & Rezazadeh, 2023). Equally important is how learners attribute their own success: when progress is attributed to their own efforts and abilities, self-efficacy will increase; when success is attributed to external factors, self-efficacy will be suppressed. This attribution logic constitutes the motivation dimension of the model: feedback type is expected to influence writing motivation and self-efficacy through motivation assessment and through the extent to which feedback provides a recognized source of self-efficacy.

Recent theoretical frameworks on GenAI in L2 writing place the above two paths within a rapidly changing feedback ecosystem. In theory, GenAI-feedback has three distinctive affordances: it is comprehensive and detailed across multiple writing dimensions, it is immediate, and it is delivered without interpersonal judgment (Dai et al., 2023; Meyer et al., 2024; Mun, 2024). These affordances map readily onto the cognitive pathway. More detailed, wide-ranging feedback should make problems in a draft easier to notice and act on, so GenAI-feedback may be particularly effective in supporting revision (Meyer et al., 2024). They map less readily onto the motivational pathway. Immediacy and the absence of judgment may reduce writing anxiety, but without a human provider, learners lose the relational and persuasive channels through which teacher-feedback has traditionally built self-efficacy (Holmes et al., 2022; Hyland & Hyland, 2006; Selwyn, 2019). Furthermore, the teaching value of GenAI depends on the interaction mode between learners and AI. Although passive reception of static feedback can provide cognitive affordability, it lacks the specific conversational participation, questioning and support for interactive use (Kasneci et al., 2023; Liu et al., 2026; Ng et al., 2025). As this study deliberately presents GenAI-feedback in a static and non-interactive way to match the non-interactive nature of teachers’ feedback, it constitutes a conservative test of GenAI’s motivational potential.

Figure 1 integrates these clues into a dual-path model. On the cognitive path, the type of feedback influences feedback processing (attention, understanding, and processing depth), which in turn shapes article revision and ultimately affects the performance after writing through internalization. On the motivational path, the type of feedback affects motivational evaluation (perception of competence, autonomy and association, as well as success attribution), which in turn activates the source of self-efficacy and ultimately influences writing motivation and self-efficacy.

The model also predicted why motivational outcomes might not automatically emerge as cognitive performance improves. First, the two paths involve different constructs and different time processes: modifying performance is a short-term, task-specific achievement, while self-efficacy and motivation are relatively stable self-beliefs that accumulate gradually through repeated, personally attributed mastery experiences (Bandura, 1997; L. F. Yang et al., 2023). Second, the two paths lead to different attributions: when the successful modification is achieved with the assistance of tools, learners may attribute the credit to the tools rather than their own abilities, thereby weakening the self-efficacy benefits generated by the same performance. Thirdly, the two sources of feedback occupy different positions in the motivational mechanism: teacher-feedback directly provides social persuasion and a sense of connection, while GenAI-feedback relies on reduced anxiety and efficiency, which are weaker and more distant sources of influence in persistent motivation. Therefore, this model allows for a separation phenomenon, that is, there are obvious cognitive differences in feedback types, while the motivational results show relatively no significant differentiation. It is precisely against this backdrop that the three research questions have been raised.

### 2.2. Teacher-Feedback in FL Writing: Cognitive Outcomes, Motivational Outcomes, and Learner Perceptions

FL writing requires learners to coordinate ideas, organization, language expression, and revision, making feedback consequential for both performance and sustained effort (Graham & Sandmel, 2011; Roca de Larios et al., 2001). Feedback may be direct or indirect, focused or comprehensive, and delivered by teachers, peers, or digital tools. The present study compares comprehensive indirect feedback from teachers, from GenAI, and in an alternating blended condition in which the two sources are used in successive feedback cycles rather than on the same draft.

Teacher-feedback remains a foundational practice in FL writing and can improve revision by identifying problems and directing learners’ attention to them (Ferris & Roberts, 2001; Fong et al., 2023). However, successful correction of a marked text does not necessarily demonstrate transfer to a new task: transfer requires learners to understand and generalize the feedback rather than apply it mechanically (Karim & Nassaji, 2020). Over-reliance on teacher correction may also encourage learners to adopt corrections passively without engaging with the underlying problems, and consequently to repeat similar errors in later writing (Lee, 2017). Accordingly, the present study examines both essay revision and post-writing performance.

The motivational consequences of teacher-feedback are conditional rather than uniformly positive. Specific, encouraging, learning-oriented comments can support competence beliefs, effort, and self-efficacy (Cui et al., 2022; Ruegg, 2018; Yu et al., 2025), whereas overly critical, overwhelming, or delayed feedback can threaten self-efficacy and motivation (Bitchener & Ferris, 2012; Fisher et al., 2025). The relevant question is therefore not whether teacher-feedback is motivating in itself, but whether learners experience it as understandable, supportive, and usable.

Learners’ perceptions are correspondingly mixed. FL writers generally regard their instructors as disciplinary experts and dependable sources of knowledge, and value their feedback as a means of avoiding repeated errors on subsequent tasks (Bitchener & Ferris, 2012). Evaluations differ across dimensions and individuals, however: some students find comments on content and organization most beneficial while others prioritize linguistic accuracy (Lee, 2005), and preferences diverge between indirect feedback, valued for the agency it affords during revision (Lee, 2017), and comprehensive individualized feedback with explicit explanations of errors (Dawson et al., 2019). Learners also report considerable difficulty interpreting comments they find vague, sometimes ignoring them altogether when revising (Kim, 2015), a problem most acute among writers whose proficiency is still developing (Lee, 2008).

### 2.3. GenAI-Feedback in FL Writing: Cognitive Outcomes, Motivational Outcomes, and Learner Perceptions

Generative AI (GenAI) systems such as ChatGPT use large language models to generate text in response to prompts (Brown et al., 2020; Chang et al., 2024). In FL writing they can deliver immediate, individualized comments across multiple dimensions of a draft, including organization, content, language use, and mechanics (AlGhamdi, 2024; Lin & Crosthwaite, 2024; Meyer et al., 2024). Importantly, they can be used in two quite different ways: as a static feedback source, from which learners receive a fixed set of comments, or as a dialogic partner, with whom they question, clarify, and negotiate meaning across turns. This distinction is central to interpreting divergent findings in the literature, since the two modes place very different demands on learners and afford very different forms of support.

Research on cognitive outcomes has asked whether LLM-generated comments support both immediate revision and sustained writing ability (Dai et al., 2023; Escalante et al., 2023; Teng, 2024). Cross-sectional evidence suggests the two come apart: learners receiving ChatGPT-feedback revised more accurately than unassisted peers but performed comparably on a new writing task (Meyer et al., 2024). Longitudinal findings are inconsistent. Escalante et al. (2023) found no significant difference between ChatGPT-feedback and teacher-feedback over six weeks, whereas Liu et al. (2026) reported stronger outcomes in a twelve-week AI-assisted intervention—though that study combined feedback with grammar and vocabulary exercises, confounding feedback source with additional instruction.

A distinction that has received insufficient attention concerns the mode in which GenAI-feedback is delivered. Learners may receive a fixed set of generated comments, acting on them without further exchange, or they may engage the system dialogically, posing questions, requesting clarification and examples, and negotiating the meaning of comments across multiple turns. The two modes differ in what they afford. Static delivery resembles conventional written corrective feedback in its one-directional character: the comments are fixed rather than calibrated to the understanding a learner is developing, and a comment that is unclear remains unclear. Dialogic use introduces opportunities for elaboration and repair, and places the learner in the position of directing the exchange. This distinction maps onto the pattern of findings reported above. Studies documenting gains typically afford extended conversational engagement, as in interventions where learners questioned the system and requested further explanation during the feedback process (Liu et al., 2026; Teng, 2024), whereas those reporting no advantage on subsequent writing have generally examined single or limited exposures to static comment sets (Meyer et al., 2024). Interpreted in this light, the evidence does not support a general claim that GenAI-feedback does or does not facilitate transfer; it suggests that the mode of learner engagement may be as consequential as the source of the feedback.

The literature also contains null and less favorable findings that constrain claims about GenAI-feedback. Escalante et al. (2023) obtained their null result under conditions broadly comparable to those reported as favorable elsewhere. Other studies have documented that AI-generated feedback can be inaccurate, overly general, or poorly matched to learners’ proficiency; such feedback requires verification that less proficient writers may be unable to perform, so that inaccurate suggestions risk being implemented uncritically (Barrot, 2023; Zou & Huang, 2024). In a direct comparison, Lin and Crosthwaite (2024) found GPT-assisted written corrective feedback not consistently superior to teacher-generated feedback, with teacher-feedback proving more accurate and more precisely targeted across several error categories. These findings caution against assuming any inherent advantage for GenAI-feedback and suggest that its cognitive benefits may be largely confined to surface-level revisions that are immediately actionable within a given draft, rather than extending to the understanding that supports performance on new tasks.

Motivational evidence is similarly promising but conditional. GenAI-feedback may enhance task value, self-efficacy, and willingness to write through immediacy and a non-judgmental tone, which can reduce writing anxiety (Fisher et al., 2025; Meyer et al., 2024; Mun, 2024; Teng, 2024). Yet these benefits appear to depend on learners’ familiarity with AI, the guidance they receive, and how actively they engage with the feedback: learners holding positive attitudes toward ChatGPT nonetheless want structured guidance in using it effectively (Nguyen & Dieu, 2024), and exposure alone does not secure sustained motivational change (Liu et al., 2026). Notably, the interventions reporting motivational gains typically involve sustained conversational interaction rather than a static receipt of generated comments—a distinction that bears directly on the present study, in which feedback was delivered non-interactively.

Learner perceptions are correspondingly mixed (Steiss et al., 2024; Wu & Ho, 2025). Across contexts, learners value the comprehensiveness, efficiency, and grammatical support of GenAI-feedback while expressing concern about its reliability and the risk of over-reliance (Mun, 2024; Nguyen & Dieu, 2024; Zebua & Katemba, 2024). Comparative studies remain inconclusive: some report more favorable perceptions of GenAI-feedback on dimensions such as unity, content development, grammar, vocabulary, and mechanics, together with appreciation of its immediacy (Erisyerico & Fauzan, 2024); others find a continuing preference for teacher-feedback in perceived usefulness and ease of use (Han & Wang, 2026; Solovey, 2024); and others report balanced attitudes, with learners valuing the interpersonal support of teacher-feedback alongside the clarity, consistency, and specificity of GenAI-feedback (Escalante et al., 2023). Taken together, these findings indicate that learners’ perceptions vary across educational, cultural, and instructional contexts, and point to the potential value of combining the complementary affordances of teacher-feedback and GenAI-feedback.

On this basis, Escalante et al. (2023) suggested that alternating or integrating the two sources—referred to here as blended-feedback—may yield greater pedagogical benefit than either alone. Their learners, however, received both sources concurrently, whereas the present study alternates them across cycles, with teacher-feedback preceding GenAI-feedback. Because each cycle involves a single source, alternation does not reduce the volume of feedback learners process at any one point. Its potential value lies instead in carryover: priorities established through teacher-feedback in one cycle may shape how learners interpret the broader GenAI-feedback they receive on a subsequent essay, and prior experience of a human reader may make them less likely to treat AI-generated suggestions as ready-made solutions (Carless & Boud, 2018; Hyland & Hyland, 2006). These remain theoretical possibilities rather than established effects. Yet the field still lacks controlled comparisons examining teacher-, GenAI-, and blended-feedback simultaneously across both cognitive and motivational outcomes—a gap the present study addresses.

## 3. Materials and Methods

### 3.1. Research Design

This study adopted a mixed-methods, intact-class quasi-experimental design. Because the participating university did not permit random assignment of individual students to conditions, three intact College English classes were assigned to one of three feedback conditions: teacher-feedback, GenAI-feedback, or teacher–GenAI-blended-feedback. The study adopted a quasi-experimental design involving both quantitative and qualitative data collection methods. The quantitative data collection methods used: (1) writing prompts to assess essay revision and post-writing performance and (2) Likert-scale to examine students’ motivation and self-efficacy in writing. The qualitative method employed an open-ended questionnaire to understand students’ perceived strengths and limitations of different types of feedback.

### 3.2. Participants and Sampling

The participants were recruited from first-year students in a public university in China. All students were enrolled in a compulsory College English course. This university was selected as they assigned students with different English proficiency into different levels of College English course according to their English subject scores in the National College Entrance Examinations.

Due to the restriction of the participants’ university, randomly assigning the participants into three different feedback conditions was not permitted; hence, three intact English classes were selected. The selection of English classes was based on three criteria: (1) the three classes were taught by the same English teacher, which minimized the possible impact brought on by different teaching strategies; (2) the majority of the students in the three classes volunteered to participate so that the number of the participants in each feedback condition was similar; and (3) their English scores of the National College Entrance Examinations were comparable.

A total of 109 students participated: 36 in the teacher-feedback class, 37 in the GenAI-feedback class, and 36 in the blended-feedback class. Participants were aged 18–21 years (11.93% were male). All had studied English as a compulsory subject for nine years (Grades 3–12).

### 3.3. Instruments

#### 3.3.1. Writing Prompts

Four narrative-writing prompts were used. One prompt was used as a baseline assessment of students’ narrative writing before the quasi-experiment. Two prompts were used for students to practice writing, receive feedback, and revise using the feedback. Another prompt was used to evaluate students’ post-writing performance after the quasi-experiment. Narrative genre was purposefully selected based on two reasons after discussion with participants’ English teacher. First, the English teacher indicated that narrative was the genre that students were most familiar with in English writing, as students had received instructions about the essential elements, techniques, and features of English narrative writing before the quasi-experiment. Second, in the semester of data collection, most of the sample English texts in participants’ textbooks and writing prompts in participants’ workbook were narratives.

All four writing prompts came from students’ workbooks, which designed the topic of writing according to the reading passages in the textbook, as this allowed the new vocabulary, grammatical structures, and collocations learned in the reading passages to be used in the writing. For instance, if the reading in a unit was on Christmas celebrations in Western countries, the writing task in the workbook was “Please write a 200-word narrative essay on an interesting Chinese New Year celebration.” All four writing prompts came from the topic bank with the same difficulty level, and the students had not written on the topic before in their English classes.

The prompt equivalence was not established empirically, for example, through independent expert ratings of difficulty or an analysis of task characteristics. Comparability across the four prompts therefore rests on their shared source, genre, target length, and the difficulty level assigned by the workbook designers. However, because the four prompts were administered in the same order to all three conditions, any residual differences in prompt difficulty would have affected all conditions equally and cannot account for between-condition differences.

#### 3.3.2. Feedback Implementation

To ensure the appropriateness of the feedback, the researchers consulted with the English teacher and agreed to adopt a comprehensive yet indirect feedback approach. The comprehensive feedback addressed both content and language aspects of students’ writings across four dimensions: organization, content, language use (e.g., grammar, vocabulary, and collocation), and mechanics (e.g., spelling, punctuation, and capitalization). The indirect feedback approach meant that the English teacher did not directly correct students’ errors; instead, the teacher provided error indications and metalinguistic clues to help students identify and revise the errors independently.

To maintain consistency between teacher-feedback and ChatGPT-feedback, GPT-4 (OpenAI, 2023) was instructed to generate indirect feedback that included only error indications and metalinguistic cues while covering the same four dimensions described above. The feedback was presented in a concise tabular format, with suggestions for each aspect limited to approximately 30 words in order to minimize students’ cognitive load (the complete prompt is provided in Appendix A). The prompt also specified contextual information about the target learners, identifying them as first-year Chinese university EFL learners. Due to restrictions on accessing ChatGPT in China, the research team generated ChatGPT-feedback for each student using an identical prompt and distributed the feedback in Word format. Although this arrangement limited students’ opportunities to interact directly with ChatGPT, it replicated the non-interactive nature of teacher-feedback.

We did not, however, formally quantify the total length of the feedback or the number of problems flagged in each condition. Although the shared protocol was designed so that the conditions differed primarily in feedback source rather than in length or coverage, residual differences in feedback quantity cannot be entirely ruled out. This is acknowledged as a limitation in Section 6.2.

In the blended-feedback condition, students received teacher-feedback on the first revision (Week 3) and GenAI-feedback on the second revision (Week 5). This fixed teacher → GenAI sequence was chosen to reflect a common classroom pattern in which the teacher first provides instructional guidance and learners then draw on AI tools. Placing teacher-feedback in the earlier cycle allowed the teacher to establish revision priorities that learners could carry into the following cycle, in which GenAI-feedback provided broader multidimensional coverage of a new essay.

We acknowledge that a counterbalanced design, adding a second blended class that received GenAI-feedback followed by teacher-feedback, would have allowed the effect of the feedback source to be separated from that of sequence, including any order-related differences in cognitive load and engagement. Counterbalancing was not feasible in this setting, however, as it would have required a fourth intact class. This would either have substantially increased the workload of the participating teacher or have required a second teacher, thereby introducing differences in instructional practice as a confounding variable. The absence of counterbalancing is therefore acknowledged as a limitation and identified as a direction for future research in Section 6.2.

#### 3.3.3. Assessment of Narrative Essays

To avoid human bias in essay assessment, all the students’ essays were graded by a web-based automated writing evaluation (AWE) system—Pigaiwang—which is widely used by Chinese higher education institutions for English writing evaluation and assessment (Z. Wang & Han, 2022). The reliability and the validity of Pigaiwang to evaluate English writing have been established by calibrating with a large corpus of human-scored English essays (Hu, 2015; X. Q. Yang & Dai, 2015). The correlations of the essay scores by Pigaiwang and human raters were high and satisfying (H. J. Wang, 2016). Blinded human double-scoring was not conducted. The implications of relying solely on automated scoring are considered in Section 5.1 and Section 6.2.

#### 3.3.4. Motivation and Self-Efficacy Questionnaire

An 11-item 5-point Likert scale questionnaire (anchors: 1 = Strongly disagree, 2 = Disagree, 3 = Neither agree nor disagree, 4 = Agree, 5 = Strongly agree) consisting of two scales: motivation in English writing and self-efficacy in English writing, were employed to assess students’ motivation and self-efficacy in English writing. The scales were adapted from the Writing Dispositions Survey (Piazza & Siebert, 2008), which were initially designed to measure native English-speaking school students’ motivation and self-efficacy in writing. To adapt them to our research contexts, the word “writing” was changed into “English writing”, and the content related to school and class was changed into College English course. As the participants’ first language is Chinese, the questionnaire was translated into Chinese for administration. A certified bilingual translator translated the adapted items from English into Chinese, and the Chinese version was then back-translated into English to check semantic equivalence with the original items. The Chinese version was reviewed by two English teachers and 10 students of a similar English proficiency level who did not participate in the study. They were asked to comment on the clarity and comprehensibility of each item. Items identified as ambiguous were revised, and the revised versions were returned for further review. This process was repeated until no further comments were received.

To verify the factor structure of the adapted questionnaire, we conducted EFA, and convergent and discriminant validity. The results of EFA show that the Kaiser–Meyer–Olkin measure of sampling adequacy was 0.72, with item-level values ranging from 0.59 to 0.84, and Bartlett’s test of sphericity was significant, χ^2^(55) = 316.98, *p* < 0.001. Parallel analysis supported a two-factor solution: the first two eigenvalues (3.41 and 1.70) exceeded the corresponding 95th-percentile values from random data (1.70 and 1.48), whereas the third (1.32) did not (1.34). A forced three-factor solution yielded an uninterpretable third factor defined by a single item, further supporting the retention of the two factors.

The two factors accounted for 36.6% of the total variance after rotation (Factor 1 = 20.6%; Factor 2 = 16.0%) and corresponded to the intended structure of the instrument. Eight items were loaded on the motivation factor (loadings 0.31–0.83) and three on the self-efficacy factor (0.53–0.97), with no cross-loadings at or above 0.30 (Table 1). The two factors were moderately correlated (r = 0.50), confirming the appropriateness of oblique rotation.

Convergent validity was measured using composite reliability and internal consistency. The results showed that composite reliability was acceptable for both factors (motivation CR = 0.76; self-efficacy CR = 0.73), as Cronbach’s alpha (motivation α = 0.78 and self-efficacy 0.71 respectively). Discriminant validity was also satisfactory: the square root of AVE exceeded the inter-factor correlation for both factors (0.54 and 0.70 versus r = 0.50), and the HTMT ratio was 0.45, well below the recommended threshold of 0.85.

#### 3.3.5. Open-Ended Questionnaire

An open-ended questionnaire requested students to list the perceived strengths and limitations of the feedback they received. The blended-feedback class was asked to provide strengths and limitations of both teacher-feedback and GenAI-feedback.

### 3.4. Ethical Considerations and Data Collection Procedure

Ethics approval was obtained from Human Ethics Research Committee at Griffith University. Data collection was in compliance with the Declaration of Helsinki. Informed consent was obtained from all the participants before the study.

**Figure 2:**
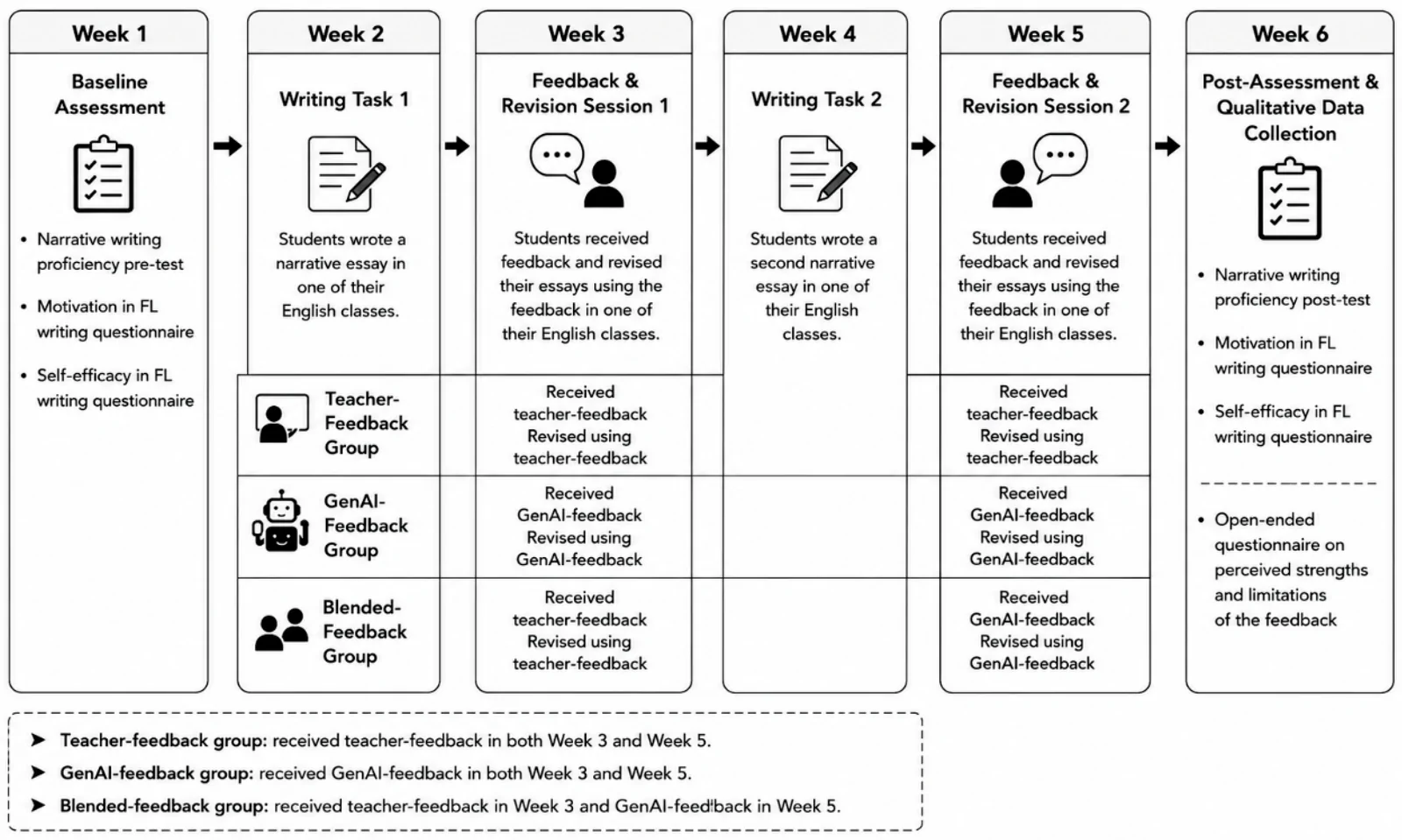
Data collection procedure.

Data collection was undertaken in participants’ College English classes spanning six consecutive weeks. In week 1 the baseline assessments of students’ English narrative-writing proficiency, motivation, and self-efficacy in English writing were conducted. In week 2, students wrote a narrative essay in one of their English classes. In week 3, students received either teacher-feedback or GenAI-feedback and revised their essays using the feedback they received in one of their English classes. Weeks 4 and 5 repeated the same procedure as weeks 2 and 3. Students in teacher-feedback and GenAI-feedback classes received either teacher-feedback or GenAI-feedback consistently in week 3 and 5, whereas students in the blended-feedback class received teacher-feedback in week 3 and GenAI-feedback in week 5. In week 6, post-assessments of students’ English narrative writing, motivation and self-efficacy in English writing were conducted. Week 6 also gathered students’ qualitative responses to the perceived strengths and limitations of the feedback via the open-ended questionnaire. The data collection procedure is presented in Figure 2.

### 3.5. Data Analysis

Quantitative data analysis was conducted using SPSS 31. Although analyses of the baseline measures indicated no statistically significant differences between the feedback groups, the corresponding baseline scores were included as covariates in the subsequent analyses to account for individual differences at pretest and to provide more precise estimates of between-group differences at post-intervention.

To address the first research question—the extent to which students’ essay revision and post-intervention narrative-writing performance differed by feedback type—a one-way MANCOVA was conducted. Feedback type was entered as the independent variable, while students’ revision and post-intervention writing performance scores were included as the dependent variables. The corresponding baseline writing-performance measures were included as covariates.

To address the second research question—the extent to which students’ motivation and self-efficacy in FL writing differed by feedback type—a second one-way MANCOVA was conducted. Feedback type was entered as the independent variable, with post-intervention motivation and self-efficacy scores as the dependent variables. The corresponding baseline motivation and self-efficacy scores were included as covariates.

To answer the third research question, concerning the perceived strengths and limitations of teacher-feedback and GenAI-feedback, students’ open-ended responses were analyzed using reflexive thematic analysis (Braun & Clarke, 2006, 2019, 2021). This approach treats coding as an interpretive act rather than a procedure for extracting pre-existing meaning from data, and treats themes as analytic outputs developed by the researcher rather than as categories awaiting discovery. Analysis proceeded recursively rather than linearly through six phases: (1) familiarization with the data, in which the responses were read repeatedly to gain an overall sense of their range and depth; (2) generation of initial codes, in which features describing strengths or limitations were identified and labeled in each response; (3) searching for themes, in which codes sharing a common pattern were grouped into candidate themes; (4) reviewing themes, in which candidate themes were compared and then refined, merged, or divided; (5) defining and naming themes, in which each theme was given a label capturing its core meaning; and (6) producing the report, in which each theme was presented with illustrative quotations and with its frequency and percentage of mentions.

The analysis was primarily inductive, developing codes from participants’ own formulations, while remaining informed by the study’s interest in how learners experienced each feedback source. Our interpretive position shaped this process in ways worth making explicit. Both authors are language education researchers with experience in EFL writing instruction in Chinese universities, and the second author was familiar with the instructional context of the participating classes. This familiarity was a resource for interpreting learners’ references to classroom practice, but it also disposed us toward a teacher-centered reading of comments about GenAI-feedback. Initial coding and theme development were therefore conducted by the first author, so that the first pass through the data was not anchored in that contextual knowledge, and the second author’s familiarity was brought to bear at the review stage, where it could be examined rather than assumed.

In the initial coding, the first author returned repeatedly to extracts that did not fit emerging patterns and reformulated provisional themes that initially reproduced the teacher–GenAI contrast assumed in our research questions rather than the distinctions learners themselves drew. The second author then engaged independently with the coded extracts and the provisional theme structure, and the two authors discussed points of divergence until a shared interpretation was reached. Consistent with a reflexive approach, this collaboration was undertaken to deepen and extend interpretation rather than to verify coding against a fixed scheme (Braun & Clarke, 2019).

## 4. Results

### 4.1. Baseline Comparison of English Narrative Writing, Motivation and Self-Efficacy in English Writing

Descriptive statistics for all the variables are presented in Table 2. Baseline comparison of English narrative-writing, motivation and self-efficacy in English writing was conducted by a one-way MANOVA. The results showed that there was no significant effect of feedback type on baseline English narrative-writing, motivation and self-efficacy in English writing: Pillai’s Trace = 0.067, F(6, 210) = 1.15, *p* = 0.336, η^2^ = 0.034, suggesting that the three classes had comparable levels of English narrative-writing proficiency, motivation and self-efficacy in English writing.

### 4.2. Results of the First Research Question—The Extent to Which Essay Revision and Post-Writing Performance in English Narrative Essays Differed by Feedback Type

Prior to conducting the MANCOVA, the assumptions of normality and equality of covariance matrices were examined. Shapiro–Wilk tests indicated that the normality assumption was not fully met. Box’s M test was also statistically significant, Box’s M = 197.51, F(12, 32,216.88) = 15.65, *p* < 0.001, indicating that the assumption of equality of covariance matrices was not met. Given these departures from the assumptions and the relatively similar group sizes, Pillai’s Trace was used as the primary multivariate statistic because of its greater robustness to assumption violations.

The MANCOVA examining differences in revision and post-intervention narrative-writing performance among the teacher-feedback, GenAI-feedback, and blended-feedback classes, while controlling for baseline writing performance, revealed a statistically significant multivariate effect of feedback type, Pillai’s Trace = 0.281, F(6, 208) = 4.90, *p* < 0.001, partial η^2^ = 0.140. Follow-up univariate tests indicated significant differences among the feedback groups for the first revision score, F(2, 105) = 6.73, *p* = 0.002, partial η^2^ = 0.129; the second revision score, F(2, 105) = 3.18, *p* = 0.046, partial η^2^ = 0.065; and post-intervention narrative-writing performance, F(2, 105) = 5.90, *p* = 0.004, partial η^2^ = 0.115.

For the first revision, the GenAI-feedback group (M = 84.58, SD = 2.65) scored significantly higher than both the teacher-feedback group (M = 75.11, SD = 6.79), *p* < 0.001, d = 0.96, and the blended-feedback group (M = 78.00, SD = 7.09), *p* = 0.046, d = 0.55. The teacher-feedback and blended-feedback groups did not differ significantly, *p* = 0.099, d = 0.41. For the second revision, both the GenAI-feedback group (M = 84.82, SD = 3.22) and the blended-feedback group (M = 83.35, SD = 4.77) scored significantly higher than the teacher-feedback group (M = 76.07, SD = 6.30), *p* = 0.028, d = 0.58, and *p* = 0.047, d = 0.49, respectively. The GenAI-feedback and blended-feedback groups did not differ significantly, *p* = 0.736, d = 0.09. Given that the blended-feedback group received teacher-feedback for the first revision and GenAI-feedback for the second revision, these findings indicate that revision scores were higher when GenAI-feedback was provided.

For post-intervention narrative-writing performance, the teacher-feedback group (M = 82.57, SD = 5.83) scored significantly higher than both the GenAI-feedback group (M = 79.02, SD = 6.03), *p* = 0.026, d = 0.59, and the blended-feedback group (M = 76.99, SD = 6.99), *p* = 0.001, d = 0.81. The GenAI-feedback and blended-feedback groups did not differ significantly, *p* = 0.426, d = 0.22.

### 4.3. Results of the Second Research Question—The Extent to Which Motivation and Self-Efficacy in FL Writing Differed by Feedback Type

Prior to the MANCOVA, the assumptions of normality and equality of covariance matrices were examined. Shapiro–Wilk tests were nonsignificant for both motivation and self-efficacy across all three feedback groups (all *p*s > 0.05), indicating no significant departures from normality. Box’s M test was also nonsignificant, Box’s M = 1.72, F(6, 195,406.88) = 0.28, *p* = 0.947, supporting the assumption of equality of covariance matrices. Thus, these assumptions were adequately met. The MANCOVA results showed there was a nonsignificant multivariate effect of feedback type, Pillai’s Trace = 0.008, F(4, 208) = 0.21, *p* = 0.935, partial η^2^ = 0.004. Follow-up univariate tests similarly showed no significant group differences in motivation, F(2, 104) = 0.20, *p* = 0.823, partial η^2^ = 0.004, or self-efficacy, F(2, 104) = 0.16, *p* = 0.854, partial η^2^ = 0.003.

### 4.4. Results of the Third Research Question—Chinese EFL Learners’ Perceived Strengths and Limitations of Teacher-Feedback and GenAI-Feedback

Themes were identified from students’ responses to the open-ended questionnaire and summarized in Table 3 with frequency and percentage included. It should be noted that the total frequencies of students’ responses did not equate to the total number of the participants, as students varied in terms of how many strengths and/or limitations they put for teacher-feedback and/or for GenAI-feedback. For the perceived strengths of the two types of feedback, thematic analysis identified six and seven themes for teacher-feedback and GenAI-feedback respectively. As to the perceived limitations of the two types of feedback, five themes were found for teacher-feedback and GenAI-feedback respectively. The details of each theme and representative quotes are presented in the following Table 3.

#### 4.4.1. Perceived Strengths of Teacher-Feedback

Six themes captured what students valued about teacher-feedback. Across these themes, perceived value was closely tied not only to the feedback itself, but also to the teacher as a knowledgeable and familiar source who understood both the learner and the instructional context.

Two themes concerned the accessibility of teacher-feedback. The first was the ease of locating problems. Students valued the concise and direct nature of the teacher’s marking, particularly when codes or annotations drew attention immediately to specific weaknesses in their writing. As one student explained, teacher-feedback “uses various forms of codes to indicate problems, so it is relatively easy for me to see my problems at a glance.” What students appeared to value here was not extensive explanation, but the ability to identify quickly where attention was needed. A related theme was the comprehensibility of language. Students frequently described the wording of teacher-feedback as accessible and easy to process; as one participant put it, “the English used in the teacher-feedback is easy for me to understand.” Together, these themes suggest that students valued teacher-feedback because it reduced the effort required both to identify problems and to understand the feedback provided.

The third theme concerned accuracy and authority. Students tended to regard teacher-feedback as trustworthy because it came from a source they associated with disciplinary expertise and legitimate evaluative authority. One participant remarked, “Teachers’ words have some authoritative elements. I feel teacher-feedback is more accurate than any other feedback.” This perception of accuracy was therefore closely intertwined with students’ confidence in the teacher’s expertise and professional role.

The remaining three themes reflected the relational nature of teacher-feedback. Emotional and human elements were valued because they made the feedback feel supportive and personally meaningful. One student described the teacher’s feedback as “full of emotions,” adding that it “could sustain my efforts in English writing and made me feel supported and encouraged in the writing process.”

Alignment with students’ proficiency was similarly grounded in the teacher’s familiarity with individual learners. As one participant explained, “My teacher knows my English writing ability, hence, the feedback she gives to me is always appropriate to my level”. Students therefore perceived teacher-feedback as being calibrated to what they were currently capable of understanding and improving. Finally, connection to textbook knowledge reflected the teacher’s knowledge of the formal curriculum. One student noted that “teachers are often able to link the problems in my English writing to the knowledge points in the textbook”. This connection helped students interpret feedback in relation to what they had already learned in class.

Taken together, these themes suggest that the perceived strengths of teacher-feedback extended beyond the content of the comments themselves. Students valued its clarity and accessibility, but they also valued qualities that depended on the teacher’s accumulated knowledge of the learner, the curriculum, and the broader teaching context.

#### 4.4.2. Perceived Strengths of GenAI-Feedback

Seven themes captured students’ perceptions of the strengths of GenAI-feedback. In contrast to teacher-feedback, which was valued largely for its contextualized and relational qualities, GenAI-feedback was primarily appreciated for its functional characteristics, particularly its breadth, speed, consistency, and efficiency.

The most prominent theme was comprehensiveness. Students valued the capacity of GenAI-feedback to address multiple dimensions of writing within a single response. One participant explained that “GenAI-feedback is comprehensive, consisting of grammar, vocabulary, collocation, coherence, and even punctuation”, adding that comments on coherence helped raise awareness of how sentences and paragraphs should be connected. Students therefore appeared to value GenAI-feedback for extending beyond local language errors to include broader aspects of organization and textual quality.

The second theme was speed and timeliness. Students frequently contrasted the immediacy of GenAI-feedback with the time required for teachers to respond to a large number of essays. As one participant observed, “Every time we submit our essays, GenAI can give us feedback quickly, but it takes the teacher at least a week to do the same thing”. This immediacy was particularly valued in the context of large classes, where students recognized that teacher-feedback was constrained by workload.

Two themes reflected students’ perceptions of GenAI as a non-human evaluator. The first was objectivity. Some students believed that GenAI-feedback was less vulnerable to emotion or personal bias, with one participant stating that “GenAI-feedback is objective, which is less likely to be affected by human emotions and bias”. The second was accuracy. Students often linked perceived accuracy to the technological scale and data resources underpinning GenAI. One student explained, “As GenAI makes use of big data to mark our essays and to provide feedback, I believe it is quite accurate”. These perceptions indicate that students associated technological processing with consistency and reliability, even though concerns about accuracy also appeared among the reported limitations of GenAI-feedback.

A further strength was the standardized format of GenAI-feedback. Students appreciated the consistency of its presentation, which made the feedback predictable and easier to navigate. As one participant commented, “I like the consistent format of GenAI-feedback. The format remains the same across every aspect of writing”. This consistency distinguished GenAI-feedback from the more variable forms that teacher-feedback could take.

The final two themes extended beyond the immediate quality of the feedback itself. Technology-supported learning reflected students’ positive attitudes toward using advanced technology in the learning process. One participant stated, “When I receive the feedback from ChatGPT on my writing, I feel that my English learning is supported by modern technology. This makes me feel positive about English learning”. Here, the perceived benefit might derive partly from the experience of learning with an advanced technological tool. Reduced teacher workload reflected students’ awareness of the potential benefits for teachers. One student noted that “ChatGPT definitely can save time for our English teacher and reduces her burden to mark our essays”.

Overall, students valued GenAI-feedback primarily for what the technology enabled them to do efficiently and consistently. Its perceived strengths were associated with breadth, immediacy, standardization, and reduced dependence on teacher time, rather than with knowledge of the individual learner or classroom context.

#### 4.4.3. Perceived Limitations of Teacher-Feedback

Five themes captured students’ perceptions of the limitations of teacher-feedback. Most of these limitations were associated with the fact that teacher-feedback depends on human time, attention, judgment, and modes of delivery.

The most prominent limitation was slowness and lack of timeliness. Students recognized that providing individualized feedback required teachers to read and respond carefully to a large number of essays. As one student observed, “Teachers are not machines, they are definitely slow in marking our writing. It takes a long time for them to complete marking all the essays and give every student some feedback”. The concern was therefore not necessarily dissatisfaction with the teacher, but with the practical delay inherent in teacher-generated feedback.

A related theme was lack of comprehensiveness and systematicity. Some students felt that teacher-feedback did not always identify all of the problems in their writing or address all dimensions of the text. One participant commented, “I found that sometimes my English teacher missed some problems in my writing”. Others suggested that teacher-feedback could lack an explicit and systematic framework. This theme suggests that students sometimes perceived the selectivity of teacher-feedback as a limitation, particularly when contrasted with the broader coverage of GenAI-feedback.

The third theme was subjectivity. Students recognized that teachers may differ in their interpretations of writing quality and in the standards they apply. As one participant explained, “Every teacher may have their own standards and unique perspectives as to what a good essay looks like. Therefore, it is unavoidable that the feedback given by teachers has subjective elements”. Importantly, students tended to frame subjectivity as a general characteristic of human judgment rather than as a criticism of a particular teacher.

The remaining two themes were more practical in nature. Difficulty reading handwritten feedback arose when students could not easily decipher written comments. One participant stated, “As the teacher provides feedback by handwriting, it is sometimes difficult for me to read her comments”. The final theme, limited flexibility, concerned access to feedback. Students noted that teacher-feedback could not always be obtained at the point when they needed it, with one participant observing that it “cannot be accessed immediately, hence, lacks flexibility”.

Taken together, these limitations reveal an important contrast with the strengths attributed to GenAI-feedback. The dimensions on which teacher-feedback was perceived as weaker—speed, breadth, consistency, and accessibility—were precisely those on which students tended to value GenAI-feedback.

#### 4.4.4. Perceived Limitations of GenAI-Feedback

Five themes captured students’ reservations about GenAI-feedback. Across these themes, a central tension emerged: although GenAI-feedback was valued for its breadth and efficiency, students often experienced it as insufficiently responsive to their individual needs and learning context.

The most prominent limitation was generic and formulaic feedback. Students frequently described GenAI-feedback as broad, standardized, or insufficiently tailored to their own level and specific writing problems. One participant explained, “ChatGPT only gives very general and broad feedback, which does not take my level of English proficiency into account”. Students therefore perceived a gap between the amount of feedback produced and its degree of personal relevance. In this respect, the comprehensiveness of GenAI-feedback could become a limitation when students struggled to identify which comments were most important for their own revision.

The second theme concerned the difficulty of the language used in GenAI-feedback. Students reported that long and complex explanations could make the feedback cognitively demanding to process. One student stated, “The language in GenAI-feedback contains long and complicated sentences, which are very difficult to comprehend”. This theme contrasts directly with the perceived linguistic accessibility of teacher-feedback and suggests that the usefulness of extensive feedback depends partly on whether students can readily understand it.

A third limitation was the absence of human and emotional elements. Students often contrasted the impersonal nature of GenAI-feedback with the encouragement and interpersonal connection they associated with teacher-feedback. One participant described GenAI-feedback as “just a kind of ‘feedback’”, adding that “the language is very cold and emotionless. It lacks the human elements as in the feedback provided by the English teacher”. For these students, feedback was not solely a source of information about writing quality; it also formed part of the relational experience of learning.

The fourth theme concerned uncertainty about accuracy. Although some students viewed GenAI-feedback as highly accurate, others questioned its reliability and felt that its suggestions required additional checking. One participant noted that “it still takes some time to verify the accuracy of GenAI-feedback”. This concern is particularly notable because it complicates the perception of accuracy identified among the strengths of GenAI-feedback, revealing that students held mixed and sometimes contradictory views about the reliability of the technology.

The final theme concerned potential negative implications for teaching. Some students worried that extensive reliance on GenAI-feedback could reduce teachers’ awareness of their learning progress and weaken teacher–student interaction. As one participant explained, “If the English teacher only uses ChatGPT to mark our essays, she would not be able to know if we have made some progress or which aspects in our English writing need more attention”. This concern extends beyond the immediate usefulness of GenAI-feedback and reflects students’ recognition that the process of providing feedback also enables teachers to monitor learning and adjust subsequent instruction.

Overall, the limitations of GenAI-feedback reveal that the same characteristics students valued—breadth, consistency, speed, and automation—could also constrain its usefulness when feedback became overly general, linguistically demanding, or detached from the individual learner. Students’ accounts therefore suggest that feedback was evaluated not only in terms of how much information it provided, but also in terms of how well it responded to their proficiency, learning needs, and relationship with the teacher.

## 5. Discussion

### 5.1. Different Pedagogical Roles of GenAI-Feedback and Teacher-Feedback on Essay Revision and Post-Writing Performance

Our results consistently showed that EFL learners receiving GenAI-feedback performed better on essay revision tasks, whereas students receiving teacher-feedback performed better on new-task-writing performance. This finding suggests that GenAI-feedback and teacher-feedback may serve different pedagogical functions in the writing process. Specifically, GenAI-feedback appears to be particularly effective in supporting text improvement, while teacher-feedback may play a more important role in facilitating the application of writing knowledge and strategies to a subsequent writing task. It should be stressed, however, that this subsequent task was itself a further narrative prompt within the same genre; the teacher-feedback advantage is therefore best described as a within-genre, near-transfer effect rather than as evidence of broad transfer to unfamiliar writing contexts.

The stronger performance of the GenAI-feedback class on essay revision may be explained by the comprehensive and elaborative nature of AI-generated feedback. GenAI systems such as ChatGPT are capable of generating extensive suggestions across multiple dimensions of writing, including content development, organization, vocabulary choice, collocations, and linguistic accuracy. Such comprehensiveness may provide learners with more breadth guidance for improving their drafts. In particular, AI-generated feedback often offers alternative ways of expression ideas, structural suggestions, and knowledge extension that can directly support learners in revising and refining their texts.

This interpretation is consistent with previous research highlighting the breadth of GenAI-feedback. For example, Dai et al. (2023) compared teacher-feedback and ChatGPT-feedback on university students’ assignments and found that ChatGPT tended to provide more elaborate comments, particularly through knowledge expansion and explanatory suggestions. Similarly, Guo and Wang (2024) reported that ChatGPT generated substantially larger volumes of feedback than teachers for EFL learners’ writing. They further observed that while teacher-feedback primarily concentrated on content and language-related issues, ChatGPT distributed feedback more evenly across content, language, and organizational aspects. Such balanced feedback may have prompted learners to not merely focus on surface-level language problems but also pay attention to content and structural improvements.

The advantage of GenAI-feedback in supporting text organization was also reflected in learners’ qualitative comments in the present study. Several participants indicated that AI-generated feedback helped them reorganize the structure of their essays more effectively and provided clearer directions for improving coherence and logical flow. This finding further supports the view that GenAI systems may be especially valuable during revision stages where learners need comprehensive guidance for refining existing drafts. Recent studies on GenAI-assisted writing have likewise suggested that GenAI can function as a dynamic scaffold by supporting idea generation and rhetorical organization throughout the writing process (Kohnke et al., 2023; Rudolph et al., 2023).

Despite the effectiveness of GenAI-feedback for essay revision, teacher-feedback remained more beneficial for performance on new writing tasks. This finding suggests that although AI-generated feedback may facilitate immediate text improvement, it may not automatically lead to the deeper development or transfer of writing competence required for independent writing in unfamiliar contexts. Several explanations may account for this pattern.

First, relying solely on GenAI-feedback may not fully utilize the broader pedagogical affordances of GenAI for language learning. In the present study, GenAI-feedback was primarily used as a feedback tool for revising drafts. However, GenAI systems can also support language development through a wider range of interactive learning activities, such as generating grammar and vocabulary exercises, answering learners’ questions, providing explanations and clarifications, and scaffolding idea development. The absence of these broader learning interactions may have limited the extent to which learners developed transferable writing knowledge and strategies. Supporting this interpretation, Song and Song (2023) found that Chinese EFL learners who participated in semester-long AI-assisted academic writing instruction outperformed those receiving traditional teacher-led writing instruction. Importantly, the AI-assisted group did not merely receive GenAI-feedback on their writing; they also actively engaged in questioning, clarification-seeking, language exercises, and interactive learning activities throughout the semester. Similarly, Teng’s (2024) study allowed students to interact with GenAI in the process of obtaining feedback, including posing questions on different aspects of writing, asking for clarifications, and seeking further information. This suggests that the educational value of GenAI may depend not only on feedback provision itself, but also on how learners interact with AI as a broader learning support system. The recent literature has also emphasized that the educational value of GenAI depends heavily on the quality of learner–AI interaction and the extent to which AI is integrated into active learning processes rather than being used merely for correction or answer generation (Kasneci et al., 2023; Ng et al., 2025).

Moreover, learners interacting with GenAI-feedback may focus more heavily on implementing suggested revisions within a specific draft, potentially leading to successful task completion without fully understanding the underlying linguistic or rhetorical principles behind them. As a result, improvements achieved during revision may not necessarily translate into independent writing competence in subsequent tasks. This interpretation resonates with concerns in the recent GenAI literature regarding learners’ overreliance on AI tools and the possibility that AI may encourage superficial engagement with learning tasks if not accompanied by sufficient reflection and instructional scaffolding (Kasneci et al., 2023; Tlili et al., 2023; Zawacki-Richter et al., 2019).

The better performance on post narrative writing obtained in our study is broadly consistent with the literature emphasizing the role of teachers in providing contextualized, pedagogically grounded feedback that learners can carry over to later tasks (Hyland & Hyland, 2006). Because the post-test remained within the same narrative genre, this pattern is best read as suggestive of near transfer rather than as conclusive evidence of transfer to unfamiliar writing situations.

Unlike GenAI systems, teachers are more capable of tailoring feedback to learners’ developmental needs, linking students’ writing problems with the current knowledge points in the textbooks, and scaffolding higher-order cognitive and metacognitive strategic writing strategies that can be transferred across tasks. Teacher-feedback may therefore promote deeper internalization of knowledge points rather than task-specific correction alone. Such guidance may also encourage learners to reflect more deeply on their writing processes and develop adaptable writing strategies that can be transferred across tasks. In this sense, teacher-feedback may better support transfer, where learners apply acquired knowledge and strategies to unfamiliar writing situations.

However, these explanations must be weighed against several alternative explanations that cannot be completely excluded from the current design. First, because the study compared intact classes rather than randomly assigned individuals, class-level factors such as classroom dynamics, peer interaction, and the placement of the course within the teaching week remain confounded with feedback condition, and covariate adjustment cannot remove these influences. The fixed teacher → GenAI sequence in the blended condition introduces a further confound, since order, timing, and novelty effects cannot be separated from the feedback source within that group.

A further concern relates to measurement. Writing performance was scored by Pigaiwang, an AWE system, rather than by human raters. AWE systems are calibrated against large human-scored corpora, but they tend to reward syntactic complexity, lexical sophistication, and surface accuracy, which are precisely the features GenAI-feedback most directly targets. The revision advantage associated with GenAI-feedback may therefore partly reflect an alignment between the features its comments address and those rewarded by Pigaiwang, rather than a corresponding improvement in overall writing quality. Because blinded human double-scoring was not conducted, the magnitude of this potential bias cannot be estimated. This does not invalidate the comparison, since all three conditions were scored by the same system under identical procedures, but it does mean that the revision findings should be regarded as system-specific until they are replicated with human raters. Future research should combine automated scoring with blinded human ratings, ideally supplemented by analytic measures of accuracy, complexity, and fluency, before the revision advantage of GenAI-feedback can be treated as conclusive.

### 5.2. Non-Significant Effect of Feedback Type on Motivation and Self-Efficacy in FL Writing

Our findings showed that feedback type did not significantly influence students’ motivation or self-efficacy in English writing following the quasi-experiment, suggesting that learners receiving teacher-feedback, GenAI-feedback, and blended-feedback demonstrated comparable levels of motivation and confidence in FL writing. This finding contrasts with several previous studies reporting positive effects of GenAI-supported instruction on learners’ motivation and self-efficacy. For example, Teng (2024) found that university EFL learners who engaged in GenAI-assisted writing instruction over one semester demonstrated significantly higher levels of writing motivation and self-efficacy than those receiving traditional teacher-led instruction. Similarly, Liu et al. (2026) reported that primary EFL learners participating in a ChatGPT-supported Cognitive Academic Language Learning Approach intervention showed significantly greater improvements in English writing motivation and self-efficacy compared with learners receiving traditional instruction alone.

However, the inconsistency between our findings and previous research may suggest that the motivational and self-efficacy benefits reported in earlier studies were not generated by feedback alone, but rather by the broader interactive and pedagogical affordances of GenAI-supported learning environments. In the present study, learners did not actively interact with GenAI systems; instead, they only received feedback generated by GenAI on their writing. By contrast, Teng’s (2024) study allowed students to engage dialogically with GenAI during the feedback process by asking questions about writing, requesting clarification, and seeking additional explanations and examples. Likewise, in Liu et al.’s (2026) study, GenAI was integrated into multiple aspects of instruction, including supplementing teaching materials, providing translations into learners’ native language, generating personalized assignments tailored to students’ proficiency levels, delivering real-time feedback, and supporting students’ self-regulated learning strategies.

These richer forms of interaction and instructional support likely provided learners with substantially greater opportunities for active engagement, mastery experiences, and personalized scaffolding, which are important sources of self-efficacy development according to social cognitive theory. In other words, the motivational and confidence-related advantages observed in earlier studies may have stemmed less from receiving AI-generated feedback itself and more from learners’ sustained interaction with GenAI as an adaptive learning partner. Compared with merely receiving static feedback, engaging in iterative dialog with GenAI may foster greater learner autonomy, perceived competence, and control over the learning process, thereby strengthening both motivation and self-efficacy.

The motivation-neutral findings can also be interpreted through the lens of self-determination theory (SDT). SDT proposes that motivation is fostered when the psychological needs for autonomy, competence, and relatedness are supported. From this perspective, an important distinction may lie not simply in whether feedback is generated by GenAI, but in how learners are able to engage with it. In the present study, GenAI-feedback was generated offline by the research team and delivered to learners in a Word document. Learners therefore could not question the feedback, request clarification or examples, ask for alternative explanations, or otherwise shape the interaction around their emerging needs.

Such static delivery may offer relatively limited support for autonomy because learners have little control over the feedback process beyond deciding how to respond to the comments they receive. Support for competence may also be constrained because the feedback cannot be dynamically adjusted in response to learners’ understanding or difficulties. Likewise, the absence of an interactive interlocutor limits opportunities for the responsive exchange that may contribute to a sense of interpersonal connection or support. By contrast, conversational use of large language models allows learners to ask follow-up questions, seek clarification, request examples, and progressively refine the assistance they receive. Such interaction has greater potential to support autonomy and competence and, depending on how the interaction is experienced, may also provide some of the responsiveness associated with relatedness. Accordingly, the absence of significant differences in motivation and self-efficacy in the present study may be interpreted as suggesting that motivational benefits are not inherent in GenAI-generated feedback itself. Rather, such benefits may depend partly on whether GenAI is used interactively and responsively, enabling learners to exercise greater agency and obtain support that adapts to their developing needs.

A further consideration concerns the duration of the intervention. The present study involved a relatively short-term treatment, whereas previous studies reporting significant motivational and self-efficacy gain typically implemented GenAI-supported instruction over substantially longer periods. For instance, Teng’s (2024) intervention was conducted over an entire semester, allowing learners extended opportunities to familiarize themselves with GenAI tools, develop confidence in using them, and gradually internalize positive learning experiences. Similarly, Liu et al.’s (2026) intervention involved sustained integration of ChatGPT into multiple instructional activities over time. Other recent studies have also employed longer-term implementations of GenAI-supported learning. For example, Song and Song (2023) integrated AI-assisted academic writing instruction across a semester and found improvements not only in writing performance but also in learners’ engagement and confidence in writing.

In contrast, the shorter duration of the present study may not have provided sufficient time for changes in motivational constructs such as motivation and self-efficacy to emerge. Motivational development often occurs more gradually than improvements in immediate task performance, particularly when learners are adapting to unfamiliar technologies and learning environments (Aldeeb et al., 2024). Research on technology-enhanced learning has suggested that while cognitive and performance gains may emerge relatively quickly, motivational constructs such as motivation, self-efficacy, and learning confidence typically require longer periods of sustained engagement and positive mastery experiences to develop (L. F. Yang et al., 2023).

Learners may initially focus on understanding how to use GenAI tools effectively before developing stronger feelings of competence, confidence, and motivation. Therefore, while short-term exposure to GenAI-feedback may support revision performance, longer-term and more interactive engagement with GenAI may be necessary to produce measurable improvements in learners’ motivation and self-efficacy.

The findings of the present study therefore suggest that GenAI-feedback alone may be insufficient to substantially influence motivational dimensions of FL writing especially within a relatively short intervention period. Instead, the motivational benefits of GenAI may depend on how deeply AI tools are integrated into broader pedagogical activities that promote interaction, personalization, reflection, and self-regulated learning.

Finally, the null motivational finding must be weighed against the temporal limits of the design. The intervention spanned a single six-week teaching cycle within one compulsory College English course. This length was fixed at the design stage by the course structure and by the requirement that all three conditions run in intact classes within the same teaching cycle; it was not chosen after the results were known. It was nevertheless foreseeable at that stage that six weeks might be too short for the motivational outcomes we set out to measure. Social cognitive theory holds that self-efficacy builds through repeated, self-attributed mastery experiences (Bandura, 1997), yet the intervention provided only two feedback–revision cycles. Including motivation and self-efficacy as outcomes within this window was therefore a design-level limitation: the study could detect only relatively rapid differences between conditions, and a null result obtained under these conditions has limited evidential value.

This limitation creates a tension with the interpretations offered above. The non-interactive delivery of GenAI-feedback, the short duration, and the attributional account outlined in Section 2.1 are each compatible with the null result, but the design cannot distinguish between them, and none was tested directly. The descriptive data further caution against attributing the null result to duration alone. Mean motivation rose in all three conditions over the six weeks (Table 1), which suggests that motivational change was possible within this period but did not differ by feedback source. We therefore interpret the motivational finding as inconclusive rather than as evidence that feedback source has no motivational effect. The design supports reasonably confident claims only about short-term revision and near-transfer performance. Claims about motivational development require longitudinal designs with longer and better-controlled exposure, capable of testing whether the motivational gains reported in semester-long studies (Liu et al., 2026; Teng, 2024) emerge only after sustained engagement.

### 5.3. Perceived Strengths and Limitations of Teacher-Feedback vs. GenAI-Feedback

Students perceived both teacher-feedback and GenAI-feedback as possessing distinct strengths and limitations, suggesting that the two forms of feedback provided different but potentially complementary forms of support in EFL writing. Teacher-feedback was primarily valued for its clarity, accessibility, and human-centered nature. Learners appreciated that teachers used concise and easy-to-understand comments that enabled them to quickly identify problems in their writing. Students also perceived teacher-feedback as personalized and aligned with their proficiency levels, reflecting teachers’ ability to adapt feedback according to learners’ developmental needs. Importantly, many students emphasized the emotional and interpersonal dimensions embedded in teacher-feedback, describing it as supportive, encouraging, and emotionally engaging. These findings reinforce the previous literature emphasizing the dialogic, adaptive, and relational nature of teacher-feedback (Carless & Boud, 2018; Hyland & Hyland, 2006). However, students also identified limitations of teacher-feedback, particularly its slow delivery, limited comprehensiveness, and potential subjectivity, which may reflect practical constraints associated with large EFL classrooms and teachers’ workload.

In contrast, GenAI-feedback was mainly appreciated for its comprehensiveness, consistency, and efficiency. Students reported that GenAI-feedback simultaneously addressed multiple aspects of writing, including grammar, vocabulary, coherence, organization, and mechanics, while also providing detailed explanations regarding text structure and logical flow. The speed and timely nature of GenAI-feedback were also perceived as major advantages, especially in contexts where rapid individualized feedback is difficult for teachers to provide. Some learners additionally associated GenAI-feedback with objectivity, technological sophistication, and standardized presentation formats that facilitated readability. These findings support previous studies suggesting that GenAI systems can provide broader and more elaborative feedback across multiple writing dimensions than human teachers (Dai et al., 2023; Guo & Wang, 2024).

Nevertheless, students also expressed substantial concerns regarding GenAI-feedback. The most frequently mentioned limitation was that the feedback was often too general, formulaic, and insufficiently personalized, making it difficult for learners to identify the most important problems in their writing. Some students also found the language used in GenAI-feedback overly long and difficult to comprehend. In addition, many learners perceived GenAI-feedback as emotionally detached and lacking human connection. This finding echoes broader concerns in the AI-in-education literature that although GenAI systems may provide efficient cognitive support, they cannot fully replicate the relational and motivational dimensions of human teaching (Holmes et al., 2022; Selwyn, 2019). Students further worried that excessive reliance on GenAI-feedback might reduce teacher–student interaction and negatively affect teachers’ understanding of students’ learning progress. Overall, the findings suggest that students viewed teacher-feedback and GenAI-feedback as offering complementary affordances, with teacher-feedback providing emotional and personalized support and GenAI-feedback offering efficient and comprehensive assistance for writing revision.

## 6. Conclusions

### 6.1. Pedagogical Implications

The findings of the present study carry several important pedagogical implications for EFL writing instruction in the GenAI era. First, the results suggest that GenAI-feedback and teacher-feedback should not be viewed as competing alternatives, but rather as complementary forms of support with different instructional affordances. GenAI-feedback appears particularly useful for supporting revision processes by providing comprehensive and multidimensional feedback on grammar, vocabulary, coherence, and organization. Teachers may therefore strategically use GenAI tools during drafting and revision stages to provide learners with extensive feedback that would otherwise be difficult to deliver in large EFL classrooms.

At the same time, our findings reinforce the continuing importance of teacher-feedback in facilitating deeper learning and transfer of writing competence to new tasks. Since teacher-feedback was found to better support new-task-writing performance, teachers should continue to play a central role in scaffolding higher-order writing strategies, fostering metacognitive reflection, and providing contextualized feedback that connects students’ writing problems with key knowledge points and learning objectives in the textbooks and curriculum.

Rather than relying solely on AI-generated corrections, teachers may help students interpret, prioritize, and critically evaluate feedback in ways that promote long-term writing development.

In addition, the findings of our study also suggest that the educational value of GenAI depends heavily on how it is integrated into broader pedagogical activities. Merely providing students with static AI-generated feedback may not be sufficient to enhance motivation or self-efficacy in FL writing. Instead, learners may benefit more from interactive uses of GenAI that involve questioning, clarification-seeking, idea exploration, and personalized language support. Teachers may therefore design writing activities that encourage students to engage dialogically with GenAI systems rather than passively accepting AI suggestions. Such interaction may help foster learner autonomy and deeper engagement with the writing process.

Furthermore, the qualitative findings highlight the need to maintain the human and emotional dimensions of feedback in AI-supported learning environments. Students valued teacher-feedback for its emotional support, contextualization, and personalization, whereas GenAI-feedback was often perceived as impersonal and emotionally detached. This suggests that teachers should continue to provide relational and motivational support even when AI tools are incorporated into writing instruction. Blended-feedback approaches combining the efficiency of GenAI with the emotional and pedagogical support of teachers may therefore be particularly effective.

Our study results also imply that students require guidance in developing AI literacy and feedback literacy skills. Since our participants frequently reported difficulties interpreting lengthy, formulaic, and sometimes overly general GenAI-feedback, teachers may need to explicitly teach students how to critically interpret AI-generated comments, identify relevant suggestions, and apply feedback selectively and effectively. Helping students understand both the affordances and limitations of GenAI-feedback may reduce overreliance on AI systems and encourage more reflective engagement with feedback.

Finally, the findings suggest that institutions and educators should adopt balanced approaches toward GenAI integration in EFL writing instruction. While GenAI can reduce teachers’ workload and increase feedback efficiency, excessive reliance on AI-generated feedback may weaken teacher–student interaction and reduce teachers’ awareness of students’ learning needs and progress as indicated by our qualitative findings. Effective implementation of GenAI in writing education should therefore aim to augment rather than replace human teaching and feedback practices.

### 6.2. Limitations and Directions for Future Research

The present study contributes additional empirical evidence to the growing literature comparing GenAI-feedback and teacher-feedback in relation to both cognitive and motivational outcomes of FL writing. Nevertheless, caution should be exercised when generalizing the findings to broader populations due to several limitations of the study. First, as discussed in Section 5.2, the intervention spanned a single six-week teaching cycle. This duration may not have allowed the repeated mastery experiences on which self-efficacy development depends, and the null motivational result should therefore be treated as inconclusive rather than as evidence that feedback source is motivationally inert. Future research should implement longer interventions, preferably over an entire semester or academic year, in order to examine the longer-term effects of teacher-feedback, GenAI-feedback, and blended-feedback on cognitive and motivational outcomes.

Second, the blended condition used a fixed teacher → GenAI sequence, so the effects of feedback source cannot be separated from those of order, timing, and novelty. Future research should counterbalance the sequence, ideally with two blended classes receiving the two orders, to establish whether the order in which sources are combined matters pedagogically. Third, although both teacher-feedback and GenAI-feedback followed an identical feedback protocol, the total length of the feedback and the number of problems flagged were not formally quantified, so residual differences in feedback quantity cannot be entirely ruled out. Future studies should record and report these quantities so that source and volume can be disentangled.

Last, all essays were scored by an automated writing evaluation system (Pigaiwang). Because automated systems tend to reward syntactic complexity, lexical sophistication, and surface accuracy, the revision advantage observed for GenAI-feedback may partly reflect an alignment between the feedback and the scoring system rather than an improvement in writing quality. Future research should combine automated scoring with blinded human ratings and, where the scripts are retained, with corpus-based measures of accuracy, complexity, and fluency.

## Figures and Tables

**Table 1:** EFA of the writing motivation and self-efficacy scale (N = 112).

Item	Motivation	Self-Efficacy
AQ4	0.83	
AQ8	0.72	
AQ3	0.60	
AQ2	0.51	
AQ9	0.47	
AQ10	0.45	
AQ6	0.32	
AQ7	0.31	
AQ5 (R)		0.97
AQ1		0.67
AQ11		0.53

*Note.* Principal axis factoring with promax rotation. Loadings below 0.30 are suppressed. (R) = reverse-scored. Factor correlation = 0.50. KMO = 0.72; Bartlett’s test of sphericity χ^2^(55) = 316.98, *p* < 0.001. Total variance explained = 36.6%.

**Table 2:** Descriptive statistics.

Variable	Teacher (n = 36) M (SD)	GenAI (n = 37) M (SD)	Blended (n = 36) M (SD)
Pre-test writing (baseline)	79.29 (4.32)	78.76 (6.53)	76.83 (6.86)
Revision 1	75.11 (6.79)	84.58 (2.65)	78.00 (7.09)
Revision 2	76.07 (6.30)	84.82 (3.22)	83.35 (4.77)
Post-test writing	82.57 (5.83)	79.02 (6.03)	76.99 (6.99)
Pre-test motivation	2.57 (0.50)	2.37 (0.56)	2.52 (0.48)
Post-test motivation	2.76 (0.47)	2.79 (0.53)	2.71 (0.55)
Pre-test self-efficacy	2.49 (0.63)	2.49 (0.72)	2.41 (0.81)
Post-test self-efficacy	2.35 (0.66)	2.41 (0.66)	2.44 (0.71)

*Note.* M = mean; SD = standard deviation.

**Table 3:** Strengths and limitations of teacher-feedback and GenAI-feedback.

	Strength	n	%	Limitation	n	%
T E A C H E R - F E E D B A C K	Teacher-feedback made it easy for students to locate errors/problems in the writing.	19	28.79%	Teacher-feedback is slow, untimely, and lacks efficiency.	19	34.55%
	The language used in the teacher-feedback was easy to comprehend.	15	22.73%	Teacher-feedback is not systematic and comprehensive.	18	32.73%
	Teacher-feedback has high accuracy and authority.	13	19.70%	Teacher-feedback is not objective and often carries subjective elements.	14	25.45%
	Teacher-feedback carries emotional and human elements.	11	16.67%	The handwriting of teacher-feedback is difficult to read.	3	5.45%
	Teachers provide feedback that is appropriate to students’ writing proficiency.	6	9.09%	Teacher-feedback is not flexible.	1	1.82%
	Teacher-feedback is related to knowledge points in the textbook.	2	3.03%			
G E N A I - F E E D B A C K	GenAI-feedback is comprehensive, covering problems of many aspects in writing.	24	33.33%	The language used in the GenAI-feedback is formulaic, general and broad, which makes it difficult to locate errors/problems in writing.	25	39.06%
	GenAI-feedback is fast and timely.	16	22.22%	GenAI-feedback lacks human/emotional elements.	15	23.44%
	GenAI-feedback is objective.	9	12.50%	The language in GenAI-feedback is difficult to understand.	14	21.88%
	GenAI-feedback is highly accurate.	9	12.50%	GenAI-feedback is not always accurate.	7	10.94%
	The format of GenAI-feedback is standardized, which facilitates reading.	6	8.33%	Relying on GenAI-feedback has a negative impact on teachers.	3	4.69%
	GenAI uses large language models to provide feedback, which makes me feel that my learning is supported by advanced technology.	5	6.94%			
	GenAI-feedback reduces the human efforts to mark the writing.	3	4.17%			

*Note.* Percentages were recalculated as the number of mentions of a theme divided by the total number of mentions in that column (teacher-feedback strengths, n = 66; teacher-feedback limitations, n = 55; GenAI-feedback strengths, n = 72; GenAI-feedback limitations, n = 64).

## Data Availability

The data that support the findings of this study are available from the corresponding author upon reasonable request.

## Appendix A. Prompt for ChatGPT to Provide Feedback

The following text is an English essay written by a Chinese student learning English as a foreign language (EFL). Please provide feedback on four aspects: structure, content, language (e.g., grammar, vocabulary, and collocation), and mechanics (e.g., spelling, punctuation, and capitalization).

Present the feedback in a well-structured table with three columns. In the first column, list the four aspects, with one row devoted to each aspect. In the second column, provide suggestions for improvement for the corresponding aspect. Rather than directly correcting errors, offer only error indications and metalinguistic explanations. Each suggestion should be approximately 30 words in length. In the third column, provide three examples to guide revision for each aspect. These examples should consist of only brief prompts or cues rather than fully developed sentences.
